# Disabled and immigrant, a double minority challenge: a qualitative study about the experiences of immigrant parents of children with disabilities navigating health and rehabilitation services in Norway

**DOI:** 10.1186/s12913-020-5004-2

**Published:** 2020-02-22

**Authors:** Shahrzad Arfa, Per Koren Solvang, Berit Berg, Reidun Jahnsen

**Affiliations:** 1 Research Department, Beitostølen Healthsports Center, Sentervegen 4, 2953 Beitostølen, Norway; 2 Oslo Metropolitan University, Faculty of Health Sciences, Oslo, Norway; 3 Department of Physiotherapy, Oslo Metropolitan University, Oslo, Norway; 4 Department of Social Work, Norwegian University of Science and Technology, NTNU, Trondheim, Norway; 5 The national cerebral palsy surveillance program, CPOP, Department of Clinical Neurosciences for Children, Oslo University Hospital, Oslo, Norway; 6 Research Centre for Habilitation and Rehabilitation Models & Services, CHARM, Institute of Health and Society, University of Oslo, Oslo, Norway

**Keywords:** Immigrant parents, Children with disabilities, Healthcare system

## Abstract

**Background:**

Immigrants and their Norwegian-born children make up approximately 18% of the total population in Norway. While several studies have been conducted on immigrants’ utilization of healthcare services, immigrant families are systematically underrepresented in international studies of children with disabilities. By focusing on experiences of immigrant parents of children with disabilities navigating health and rehabilitation services in Norway, this study generated knowledge of how accessible and tailored the services were from their point of view.

**Methods:**

This study took a qualitative approach, using semi-structured interviews to explore the experiences of immigrant parents of children with disabilities from non-Western countries. The interviews were transcribed, coded, and analyzed via an inductive thematic analytic approach.

**Results:**

The findings show how the “immigrant experience” influenced the way the parents looked at, experienced, and even praised the services. The parents appreciated the follow-up services provided by the pediatric rehabilitation centers, which they experienced as predictable and well-organized. While navigating the services, they experienced several challenges, including the need for information, support, and timely help. They felt exhausted because of years of struggle in the healthcare system to access the help and services they needed. They expressed how this struggle had affected their own health. The feeling of being treated differently from the majority was another challenge they experienced while navigating the services. The findings also show how parents’ experiences of communication with healthcare providers were influenced not only by their own language and communication skills but also by the healthcare providers’ intercultural communication skills and dominant organizational culture.

**Conclusions:**

The parents’ experiences show that there is still a gap between the public ideal of equal healthcare services and the reality of the everyday lives of immigrant families of children with disabilities. By exploring immigrant parents’ experiences, this study highlights the importance of mobilization at both the individual and systemic levels to fill the current gap and provide tailored and accessible services to the entire population.

## Background

The composition of the Norwegian population, as in other countries in Europe, has been changing [1], p., 3. Immigrants and their Norwegian-born children comprise approximately 18% of the total population in Norway. More than 80% of all immigrants in Norway come from non-Western countries [2]. Immigrants, like the rest of the population, are not a homogeneous group [1], p., 4. They come from 221 different countries and independent regions for many different reasons [3]. They vary in ethnic, cultural, educational, and socioeconomic backgrounds, as well as the length of their stay in Norway [2].

An inclusive and equal society is an important goal of the Norwegian government, important both for the individual and for society. An equal healthcare service is a very important part of and condition for achieving this goal [1], p., 3. However, the consumption of healthcare services in Norway varies both within different immigrant groups and between immigrants and the general population [4].

Increasing ethnic diversity in the population and differences in the utilization of healthcare services pose challenges to the authorities’ stated goal of equitable healthcare. Improving knowledge of what influences immigrants’ use of healthcare services will therefore be beneficial for planning policies and delivering healthcare services [4]. While much research has been conducted on immigrants’ utilization of healthcare services, immigrant families are systematically underrepresented in international studies of children with disabilities [5].

A critical review of the international literature on immigrant and refugee families of children with disabilities revealed an absence of information about their access to, utilization of, and experience with community-based healthcare and rehabilitation services [6]. Bailey et al. [7] studied 100 Latino couples parenting young children with disabilities in the United States (US). They found that awareness and utilization of services varied among these families, and high awareness was associated with high use of services. Interestingly, their findings showed that none of the demographic variables, such as the education of parents, were correlated with awareness and use of services. The results revealed how parents with low awareness and utilization tended to be satisfied with the services they had received, and did not actively pursue additional information. Thus, the authors suggested that family variables such as feelings of control, which could influence an individual’s ability to gather information and use necessary services, might be more important determinants of awareness and use.

A recent review of 17 international articles revealed increased barriers to care for immigrant families of children with special healthcare needs, partly due to the difficulty in navigating a challenging and changing healthcare system [8]. A qualitative study in Canada also showed that immigrant parents of children with disabilities not only faced the same barriers as majority families but also encountered additional challenges [9]. Additionally, a narrative review of 39 articles, mostly from the US and United Kingdom (UK), showed that immigrant parents of children with disabilities mainly face additional challenges due to language barriers [10]. According to Bailey et al. [7], parents in minority ethnic groups may also face challenges due to lack of familiarity with cultural expectations of the proper way to seek help, and professionals who are not fully aware of the implications of ethnic diversity with regard to values, goals, and behavior. Fellin et al. [11] interviewed 43 clinicians within two pediatric centers in Canada. They highlighted the need for clinicians to provide culturally competent care to immigrant families of children with disabilities, by being aware of their own cultural and/or professional worldviews and the views and experiences of these families. Two UK studies of Pakistani and Bangladeshi families with a severely disabled child revealed how service providers’ stereotypical perceptions of immigrant families created challenges during these families’ encounters with the healthcare system [12, 13].

Research conducted in Norway and Denmark also showed how service providers largely explain the challenges they face while interacting with immigrant families as being the result of cultural and religious differences [14, 15]. Because of this, service providers may easily overlook other important matters and instead create stereotypical images of “the others” with reference to their culture or religion [14]. This generalization can hinder the building of individual relationships to meet the unique needs of each family. In a study of minority parents of disabled children within the Norwegian healthcare system, Söderström [16] highlighted how language difficulties and stereotypical assumptions made it difficult for minority families to access healthcare services.

The legitimacy of the healthcare system is a product of its ability to provide timely and appropriate services to the entire population [17]. Increasing ethnic diversity in the population necessitates a great deal of flexibility, creativity, and professional expertise of healthcare services to provide equal access, usage, and outcomes for the entire population [16]. Research shows that lack of service use has more to do with how the services are organized than with the characteristics of the families [5, 12, 13]. However, limited literature has told the stories of immigrant families who have children with disabilities [18]. A lack of knowledge of these families’ experiences interacting with the healthcare system limits the cultural integrity of practices within healthcare services [14, 18]. McKay [8] noted a lack of data about best practices with regard to immigrant children with special healthcare needs. According to Fellin et al. [11], formal procedures are absent for developing approaches and treatment plans based on interactions between professionals and immigrant families who have children with disabilities, despite their importance for the support of those families.

It is, therefore, important to conduct research focusing on diverse populations to enhance the further development of healthcare services and provide appropriate care and guidance to immigrant families who have children with disabilities. This study reveals the experiences of immigrant parents of children with disabilities who are navigating the Norwegian healthcare system, particularly pediatric rehabilitation services.

### The Norwegian healthcare system

The Norwegian healthcare system is publicly funded [19] and characterized by universal coverage and public provision of services [20]. It is semi-decentralized, with municipalities responsible for primary healthcare services and the state responsible for specialist healthcare services [19]. Primary healthcare includes long-term care services, general practitioners (GPs), physiotherapists, community health nurses, and emergency care. Specialist healthcare includes both private specialist healthcare providers and hospitals [21].

Residents are assigned a GP who acts as a gatekeeper to specialist healthcare services. Patients, except children and pregnant women, pay a subsidized consultation fee when visiting their GP. Most medical specialists outside hospitals, as well as outpatient hospital services, require co-payments [22]. There is also private healthcare in Norway where one can access specialists directly, but out-of-pocket fees are typically four times higher [21]. Private healthcare is relatively uncommon and mainly available in urban areas [20].

The primary and specialist healthcare systems both provide pediatric rehabilitation services, but the tasks are more specifically defined for specialist healthcare services. The primary healthcare system has the overall responsibility for follow-up, providing the necessary examination to identify the need for rehabilitation, and if necessary refer these children to specialist healthcare services. Children and adolescents from 0 to 18 years with congenital or early acquired disability, developmental disorders, or chronic illness may be entitled to pediatric rehabilitation services in the specialist healthcare system. Multi-professional pediatric rehabilitation teams have the core responsibility to provide services and follow-up to children in accordance with their overall needs. Most of the services are provided as outpatient services, but may also be provided at children’s wards in hospitals [23].

## Methods

This study was conducted with a qualitative approach, using a semi-structured interview guide (Additional file 1) to explore the experiences of immigrant parents seeking care for their children with disabilities. Parents were asked about their experiences with both primary and specialist healthcare services in general, and pediatric rehabilitation services in particular. By focusing on the experiences of immigrant parents from non-Western countries, this study generated knowledge of how accessible and tailored the services were from their point of view.

### Participants

Applying convenience sampling, this study included immigrant parents of children with disabilities from non-Western countries. The participants were recruited through a rehabilitation center between 2015 and 2017 via an information letter about the study. The potential participants who lived in the Oslo area were additionally informed about the study verbally by phone by the first author in simple language after they had received the information letter. The sample included 23 parents, with six fathers and 17 mothers who were immigrants from 14 different countries (Table 1). One potential participant declined to participate in the study, because of family difficulties.

**Table 1:** Number of participants and countries of origin

Country of origin	Number of participants
Afghanistan	1
Bosnia	1
Bulgaria	1
Chechnya	2
Iran	1
Iraq	2
Jordan	1
Pakistan	4
Poland	1
Serbia	1
Somalia	4
Sri Lanka	2
Tunisia	1
Zimbabwe	1

Some participants were familiar with the first author prior to the study commencement, due to her role in a prior developmental project intended to inform and encourage immigrant families to participate in a pediatric rehabilitation program at a Norwegian rehabilitation center with adapted physical activity as the main intervention. One of the families was already familiar with the first author due to her role as their child’s physiotherapist.

Although we used convenience sampling, the participants varied in their educational and socioeconomic backgrounds, Norwegian language skills, and length of residences in Norway (Table 2). Because the healthcare system in Norway is organized similarly across the whole country, the experiences of the services would remain largely the same regardless of the city of residence.

**Table 2:** Sociodemographic characteristics of the participants

	Sex	Age	Education	Norwegian language skills (estimated by the first author)	Length of stay in Norway (years)
1	F	30–40	University	Very good	15
2	F	40–50	High school	Good	15
3	F	30–40	Primary school	Very basic	15
4	F	30–40	High school	Very good	28
5	F	40–50	High school	Very basic	18
6	M	50–60	University	Very basic	8
7	F	40–50	University	Very good	17
8	F	30–40	High school	Good	8
9	F	40–50	Primary school	Very basic	28
10	F	40–50	Primary school	Almost none	9
11	M	40–50	High school	Basic	29
12	M	50–60	High school	Basic	12
13	M	40–50	University	Very good	29
14	F	40–50	Primary school	Good	22
15	F	30–40	High school	Basic	15
16	F	40–50	University	Basic	8
17	M	30–40	High school	Basic	7
18	F	40–50	High school	Basic	23
19	F	30–40	University	Very basic	10
20	F	40–50	University	Very good	20
21	F	40–50	University	Very good	21
22	F	30–40	High school	Very good	19
23	M	30–40	University	Very basic	4

### Data collection and analysis

Twenty-three interviews were conducted from April 2017 to January 2018. All interviews were conducted by the first author in Norwegian, except one that was conducted in English. Data saturation was discussed with the co-authors and considered achieved. The first author is an immigrant from the Middle East herself. She worked as a pediatric physiotherapist in the primary healthcare system in a multicultural district in Oslo for several years. Both as an immigrant herself and as a health professional, she was familiar with some of the challenges that the participants may have experienced while navigating the Norwegian healthcare services. The shared experiences and cultural familiarity were a foundation for building trust between her as a researcher and the participants.

Professional interpreters contributed while interviewing six participants. The use of interpreters was based on the first author’s perception of the participants’ language skills during the telephone conversation prior to the interviews, if the participants did not mention the need themselves. The interpreters explained their role and their duty regarding confidentiality, and signed a declaration form prior to each interview.

Prior to each interview, the first author explained the study purpose and regulations regarding confidentiality, then obtained written informed consent from all participants. She also emphasized that they could withdraw their consent without giving any reason if they wished to later. The participants were informed of the interview procedure and the recording of the interviews. Interviews lasted for approximately 55–130 min and were conducted at a place and time convenient for each participant, including participants’ homes, a café, Oslo Metropolitan University, the rehabilitation center, and the Family House health and educational service providers in the participants’ local districts. One of the interviews that was conducted in two different days lasted for approximately 170 min in total. While interviewing two participants, their spouses were present during parts of the interviews.

During the interviews, the first author continuously asked participants if she had understood their statements correctly, to ensure that she had captured their meanings accurately. Two participants were contacted and interviewed again by the first author to obtain further clarity about some subjects. All interviews were recorded and transcribed verbatim by the first author. Transcription of the first four interviews took place immediately after conducting the interviews, which was useful for fostering reflection on the applied terms and construction of interview questions based on participants’ understanding and responses. Then, the interview questions were modified, and new questions were added to the interview guide. However, adjustments of the interview guide were constantly made while conducting the interviews, depending on the participants’ responses and the interview context. The interview guide explored three domains: (1) the experience of services in terms of both strengths and challenges, (2) the experience of interacting with healthcare providers, and (3) the experience of receiving information. The interview guide was developed based on experiences from the former developmental project at a rehabilitation center, with informal conversations and observations of the participants who were later recruited to the present project.

The transcription of interviews was an ongoing process, as was conducting the interviews; the process of transcribing the interviews continued for some time after completing the interviews. Therefore, the first author listened to all audiotapes again right before analyzing the transcripts to refamiliarize herself with the context of each interview and the content of the data as a whole. An inductive thematic analytic approach [24] informed by interpretive description [25] was applied to explore the potential patterns in the data. Each interview transcript was read individually in an active way, searching for potential patterns, and initial data-driven coding was performed. The initial codes were defined broadly to bring together a group of data extracts that could be related. As a result, the data were ultimately organized into 17 codes, such as the strengths of the services, the experience of communication, and the interpreter. At the same time, a “quotable quotes” file was created, including particularly powerful pieces of data to ensure that they would not be lost, while also preventing them from dominating the evolving analytic process [25], p., 149. A “reflection notes” file was also created to register the first author’s reflections and thoughts while analyzing the data.

After organizing the data extracts into different codes, the analysis involved making sense of the relationships among the various groups by moving within and across the groups of data extracts within each code. A repetitive thinking and reasoning process, and shifting attention from similarities between certain cases to the differences between other cases, led to the deconstruction of the initial groups. By linking data elements together across the different codes and discarding some codes, data extracts were reorganized into six initial themes: (1) the alienation of immigrants, (2) communication between immigrant families and healthcare professionals, (3) gratitude towards the healthcare system, (4) the battle to access help, (5) access to information, and (6) prejudice as an extra challenge while navigating the healthcare system. Thereafter, the data within each initial theme were further analyzed, and subthemes were generated to give structure to the themes by elucidating the variation and details within them. For instance, three subthemes were identified within the theme “access to information”: “lack of information”, “challenges to accessing information”, and “facilitators of accessing information”. Next, the initial themes and subthemes were reviewed by all four authors to ensure that they were appropriate with regard to the dataset, that the data within the themes cohered meaningfully, and that there was a clear distinction between the themes. Subsequently, the themes were refined; one theme that did not fit with the others was discarded (“alienation of immigrants”), and two themes were combined to form a new one (“the battle to access help” and “prejudice as an extra challenge while navigating the healthcare system”). Finally, four themes were identified, and representative quotes selected to generalize the descriptions of the participants’ experiences. The first author discussed the identified themes with a non-involved peer, a physiotherapist with a PhD in qualitative research methods, which contributed to further reflections and enhancing the rigor of the final analyses.

The four themes were further interpreted in the context of previous literature. Although the different phases of the analysis are described here as being linear, the process of analysis was actually done by moving back and forth throughout the different phases.

## Results

Each participant had a unique story, and their experiences of the services and challenges were diverse. However, four main themes were identified after analyzing the data: (1) immigrants’ gratitude for the services, (2) communicating beyond language, (3) finding a way through the service system, and (4) accessing help as a battle.

### Immigrants’ gratitude for the services

The participants were mainly satisfied and grateful with regard to the services, especially the follow-up services provided by the pediatric rehabilitation centers. They experienced the follow-up services as continuous, predictable, and well-organized. They also felt safe that their children were in good hands and would receive follow-up services from caring and competent healthcare providers. Some participants were even cautious of discussing the challenges they had experienced while navigating the healthcare system because they were afraid of being perceived as ungrateful or demanding.

One participant explained how satisfied she was with her son’s checkup routines and her ability to stay in touch with his physicians over many years. She expressed her trust in and gratitude for the healthcare providers:

*They [the healthcare providers] have done the best they could do … I feel that one should not criticize and be very picky about things that are unnecessary because people who are ungrateful to humans are ungrateful to God, too. (P 9)*

A culture of gratitude and appreciation is a major part of the values that this participant, like many others in this study, grew up with and believe in. Thus, this participant, like many others, was initially hesitant to talk about what she had struggled with over the years while navigating the healthcare system.

Some participants experienced that the follow-up services had a holistic approach and included several important dimensions with regard to their children’s development. They were satisfied with how their children’s wellbeing and progress at school had been followed by the healthcare providers. Their experience was that the healthcare providers supported them in overcoming the challenges that their children faced at school by cooperating and participating in the school meetings. One participant explained his satisfaction with the services:

*I am satisfied with the follow-up services. You know for sure how the pediatric rehabilitation works; they follow the children’s health conditions, behaviors, and schooling. (P 11)*

This participant noted his appreciation of how the follow-up services comprehensively took care of his child’s needs.

The immigrant experience was highlighted when the participants praised the services. One explained that in her home country, her child could not even go to the ordinary school with the other children, and they had to pay a substantial sum of money to buy her a wheelchair.

*I'm satisfied with everything … we have different assistive devices and exercise equipment. She is riding, she is very happy, she goes to the physiotherapy, and she goes to the school, which is also important. We cooperate with the school and the hospital, and there is a lot of responsibility. (P 8)*

She deeply appreciated the services, acknowledging that her child would not have the same opportunities in their home country because of her disability. She appreciated her child’s happiness in participating in different activities like other children. Similarly, several participants compared their experiences of services in their home countries with Norwegian services, which reinforced their gratitude and satisfaction. Some appreciated being involved and informed about their children’s condition while receiving well-organized follow-up services, which was not common in their home countries.

*They always plan what the next step is going to be and how. The doctor discusses her [condition], and they inform us of what they think has to be done. We are grateful for it because not all countries give these opportunities. (P 1)*

Coming from countries in which the doctor–patient relationship is based on the idea that doctors exert professional authority through medical expertise, the participants appreciated the opportunity to be involved in the physicians’ decision-making and follow-up procedures. Some experienced being involved in this process as informative and educational. Participants also appreciated how attentive the healthcare providers were with the children and how well they handled and took care of them. One participant explained how she, as a newcomer to Norway, experienced their first meeting with the healthcare providers:

*The way they talk to the kids; the kids feel so safe here. They are not afraid. I don’t know; it was a total shock. And in the beginning, when they started talking, I wanted to cry. Because I felt like a human being, like a real human being. This was really new to me. Also when you feel that everyone will help you... I'm so grateful. (P 16)*

The participant was grateful for the way the healthcare providers approached her and her child calmly and with respect. She explained that she was very emotional in the beginning because this type of approach was unfamiliar to her, and how differently they were treated by the healthcare providers in their home country. She recalled the day that she was kicked out of the hospital because she complained about what she perceived to be wrong treatment of her child. She experienced that those healthcare providers did not listen to her at all. She explained how in their first meeting with the physician here in Norway, she did not talk because she was afraid of being treated the same way as in her home country.

The participants’ statements illustrate how the immigrant experience made them look differently at the services available to them in Norway. Comparing the services with those in their home countries made them even more appreciative of the Norwegian healthcare system, knowing that their children’s needs were being met by qualified and empathic healthcare providers.

### Communicating beyond language

The participants experienced communication with the healthcare providers differently; while some perceived it to be good or very good, others perceived it to be difficult or stilted.

Several participants experienced communication difficulties because of language barriers, especially during their first years in Norway. They experienced communicating through interpreters as challenging and emphasized how disturbing and frustrating it was to communicate through interpreters, whom they perceived as unprofessional. One participant who was dependent on an interpreter to communicate with healthcare providers shared her experience:

*Sometimes they [the healthcare providers] order a good interpreter, but sometimes they order an interpreter who misinterprets! Then, the healthcare providers misunderstand, and it bothers me for the rest of the day … It is important that they order a professional interpreter … It is about a human life. (P 10)*

The experience of being misinterpreted by unprofessional interpreters, given the importance of the content of the conversation, was disturbing to the participants. They also felt insecure about how correctly the information from the healthcare providers was relayed to them. Participants stated that they did not give feedback to the healthcare providers about their experience with the interpreters, rather relying on the hope that they would have a different interpreter the next time. However, one participant was frustrated at having the same interpreter several times in a row.

Participants also occasionally communicated with healthcare providers through their spouses or children who could speak Norwegian. Although they did not complain about that, the following experience shows how damaging it could be to communicate through relatives in the context of the healthcare system.

*They found out that the baby was not normal when they did an ultrasound [during pregnancy]; however, my husband asked them to not tell me about it because it could upset me! Also, when the baby was born, his face was different! A week after birth, the doctor told me about the baby’s condition. (P 9)*

While interviewing this participant, her husband confirmed that he knew about the baby’s condition before the birth. Although the participant justified her husband’s decision to not inform her, the fact that she had the right to know about the baby’s condition during her pregnancy is undeniable.

Other participants were satisfied with their communication with their healthcare providers despite the language difficulties. They explained how their ability to ask questions and the healthcare providers’ patience and ability to explain contributed to overcoming language barriers while communicating. A participant with basic language skills who was satisfied with the communication shared his experience:

*We had good communication. We did not have communication difficulties! But if I do not understand what they say in Norwegian, I'm not just going to pretend I understand. I have to understand what this is about... I ask them to explain. (P 11)*

Although these participants were confident about their communication with the healthcare providers, there is no guarantee that they truly understood the conversation completely. Speaking a language partially can be more challenging than not speaking the language at all. The reason for this is that neither the clients nor the healthcare providers ask for a professional interpreter, which could lead to the loss of important information.

Some participants believed that communicating in the same language was not enough to experience good communication in the context of the healthcare system. They believed that the healthcare providers’ engagement, empathic listening, and even their body language influenced the experience of the communication. A participant who could speak a little Norwegian explained what he believed about communication:

*I remember there was a professional doctor at the hospital, where we communicated through an interpreter, but it was still difficult to get to the point and communicate. Conversely, there was a social worker who was also professional, and we understood each other quite well and were on the same page, even though we did not use an interpreter... I think culture plays an important role. Possibly, the doctor has not been in touch with immigrants like me, perhaps we communicate differently, maybe they have been educated differently … (P. 6)*

This participant noted culture and experience as important dimensions of communication. To him, experiencing good communication was not only about speaking the same language but also about the healthcare providers’ intercultural communication skills, because culture and communication are strongly intertwined. One of the participants even perceived that belonging to different cultures affected the communication with healthcare providers by making both parties more cautious than they might otherwise be. Some participants also noted how the dominant organizational culture, especially time pressure, influenced communication despite management of the language barrier. A participant who spoke fluent Norwegian shared her experience related to her child’s routine checkup by a rehabilitation team:

*Even though we are physically present, mentally we are almost not there, because it is an unfamiliar language and things happen very quickly, and it is not natural to stop very often and ask them what something is, because they have to go through everything in an hour and a half … It's too short a time … The last few times, it became more like writing what they need in relation to the CPOP [The national Cerebral Palsy Surveillance Program]. It was the program that was the focus rather than what we would need in the future. (P 20)*

Time pressure, routine tasks, and the surveillance program were perceived as challenges by this participant in communicating with healthcare providers. She also experienced medical terminology as an unfamiliar language, which affected her ability to completely follow the conversation. Participants’ experiences reveal that communication in the context of the healthcare system depends not only on the communication skills of both the clients and the healthcare providers at an individual level but also on the dominant organizational culture at a systemic level.

### Finding a way through the service system

Knowledge of the law, their rights, and the services they were entitled to was important to the participants. They experienced that healthcare providers did not inform them about the rules or their rights. Furthermore, language barriers made it difficult for them to access and comprehend this type of information themselves. One participant who experienced that information was not made available to immigrants reflected on the possible reasons:

*There is no information, or if there is, it is hidden, maybe it is in Norwegian. I don’t know, but a lot of information is not readily available to immigrants, I don’t know why. Perhaps the health caregivers just assume that you know what to do or where to get the information from. (P 19)*

This participant’s statement reveals the need to make information available in different languages. Furthermore, healthcare providers need to be aware of the importance of informing and enabling immigrant families who are trying to navigate the services. Several participants also experienced that they received information randomly by meeting other parents. Some said that they received information by being associated with organizations for children with disabilities. A participant who had tried to access services to which they were entitled, through their GP, shared her experience:

*We have experienced that many healthcare providers, even our regular GP, who is absolutely fantastic, do not have information about our rights … So I think if healthcare providers who are supposed to provide information to us do not know about it themselves, it would be very random how multicultural families get access to information. (P 20)*

This participant noted the importance of being prepared as a healthcare professional to provide the information that immigrant families need to access the services to which they are entitled. Lack of information about the services available and how to access them limited the participants’ abilities to navigate the services. It was not easy for some of them to know what to ask healthcare providers. Some participants stated that the healthcare system was structured in a way that only benefited privileged clients who were already able to participate fully. They emphasized the importance of tailoring the services to different groups in society to offer accessible and equal services to the entire population. A well-educated participant who defined herself as a resourceful and well-integrated citizen noted the need to enable the immigrants she defined as disadvantaged:

*There are immigrants who have less knowledge and do not know what to ask about. They do not know which services are available, so maybe it would be good to inform them … I think society expects that this group of immigrants will integrate well, but the society is responsible for informing them. (P 7)*

This participant’s statement highlights the importance of enabling the immigrants to not only navigate the services but also integrate into society. As she noted, access to the services is an important element of integration.

Among the professionals discussed, social workers stood out. Participants met social workers mainly after their children were diagnosed. The participants were mostly satisfied with the information and support they had received from the social workers. Some noted that receiving the information was not helpful if they did not receive the guidance and help they needed to access and navigate the services:

*I was in touch with a social worker earlier; she told me about our rights, and how to access the services … I cannot write very much, like proper letters, so after receiving the necessary medical documents from the GP, I used to go to the social worker and she helped me with that. (P 15)*

As this participant noted, navigating the services demands special skills, such as an appropriate level of writing skills to apply for services. Application letters have to be well-documented and convincing. This participant used the social workers to not only obtain the information but also navigate the services. However, other participants experienced that the information provided by social workers was not comprehensive. Despite the fact that the participants’ need for information was continuous as their children grew up and entered new stages of development, their relationship with the social workers was not continuous. They stated that they met the social workers only a couple of times in the very early years after their children were diagnosed. Considering that family situation and healthcare options vary over the years, the participants’ need for information was not completely met by the social workers.

### Accessing help as a battle

Some participants described years of struggling to get help. They felt that their concerns had been underestimated and not taken seriously by the healthcare providers. They described how they consistently had to insist on their need for help, and even wondered if they had been perceived as nagging or rude. They felt exhausted and experienced their attempts to get help as stressful, frustrating, and demanding in terms of resources. They stated that the struggle over the years had affected their mental and physical health. Some participants described it as challenging to obtain access to the specialist healthcare system through GPs, who acted as gatekeepers. One participant felt that her worries about her daughter had been underestimated by their regular GP for several years:

*We really experienced from the beginning that she was very uneasy, cried a lot, and was delayed in her motor skills, so we arranged an appointment with our regular GP, and she told us that there was no reason to worry … When she turned three years old, we still experienced challenges; then we arranged a new appointment with the GP, and she still said that it was normal until she turned five years old. (P 20)*

This participant explained that they were worried because they observed that their child fell a lot. They decided to reach out to their regular GP again to obtain a referral to the orthopedic outpatient clinic. Although the GP referred their child to a specialist this time, she was not prioritized.

*...But we did not receive any response, so we called them, and they said she was not a priority... Again, we went back to our regular GP. It was already over one year later, so she sent a reminder of the referral … Then we got an appointment at the orthopedic outpatient clinic, and the physician recognized what was wrong, so she sent us to the children's department at the hospital immediately... She was five at the time. (P 20)*

Five years of being sent back and forth to access help from the healthcare system was a burden on this family. This participant described how they could not pay enough attention to their son because they were focused on getting help for their daughter. She believed that this entire burden could have been alleviated if their concern had been taken seriously by the healthcare providers in the beginning.

Another participant who described accessing help as a battle wondered whether healthcare providers perceived the parents’ concerns as excessive. She recalled how even after her child was finally hospitalized with a swollen and painful knee, it took almost two months before an orthopedist visited and referred her for further examinations. It had been several years since her child was diagnosed, but she still experienced attempts to access help through emergency care and their regular GP as continually “hitting a wall”. Another participant even generalized her experience of not being taken seriously by the doctor as a common pattern while seeking help in Norway. She further explained how years ago when her child was only 18 months old, they had to take her to emergency care three times, and each time they were sent back home. Finally, they had to take her to the hospital, where she went into a coma and was hospitalized for a month. According to this participant, the child was never the same after that, and they struggled to get the help they needed for several years.

*We used to receive an appointment with the doctor. We went there and talked to the doctor [about our child], but as you may know, he kept saying that everything is fine. As you may know in Norway, the doctors always say that everything is fine. (P 5)*

Some participants with similar experiences explained how they occasionally chose to take their children to private emergency care or specialists in order to get proper and timely help, even though they had to pay extra for the consultations.

Other participants felt that healthcare providers did not always pay attention to their concerns or were not interested in listening to their worries. They described how healthcare providers interrupted them or changed the subject of the conversation, which they might have thought to be irrelevant. One participant described worry about his family’s situation not interesting healthcare providers:

*I have experienced that it is not important for them to know or hear about my financial situation or about my residential situation, or when I am talking about transport, I have noticed that they do not care. In my opinion, our financial and residential situation is related to my children’s health, but they do not see it, they do not understand it. I have always talked about it to our regular GP... (P 11)*

This participant felt that the healthcare providers ignored and overlooked what the family truly struggled with. He believed that the healthcare providers did not consider his worries to be of interest or relevant to their jobs. He then described how exhausting it had been not to receive timely help:

*… The problem is that it takes so long, you get help when you have become completely exhausted. You have to approach them and complain again and again; they do nothing until you fall down... Then they will help you. (P 11)*

He finally described how the demanding and stressful process of getting help over the years had caused him a lot of stress and disturbed his mental balance.

Language barriers and lack of knowledge of medical conditions made it even more challenging for some participants to access timely help. One participant, whose daughter experienced seizures for several months before receiving help, shared her frustration:

*Every time I went to the hospital, I explained how she used to lose consciousness and started kicking and how disturbing it was for her. Every time I told them, they replied that it has something to do with her nerves. (P 10)*

She explained that she did not know anything about seizures, and this condition was quite unfamiliar to her. The participant’s difficulty in precisely describing her child’s condition caused significant delays in receiving a proper diagnosis and specialized services for her daughter. The participant explained how, after several months of suffering and trying to navigate the healthcare system, her daughter finally received the treatment that she needed. This family’s experience illustrates how important it is for healthcare providers to be aware of language barriers and inadequate knowledge about medical terms among immigrant families. Being curious as a healthcare provider and paying enough attention while interacting with immigrant families would prevent others from experiencing what this family went through.

When interpreting and reflecting on their experiences with healthcare services, some participants brought up their position as immigrants. They believed that the way they were treated by the healthcare providers was important. Some participants perceived that their skin color and religion influenced how they were treated and the services they had received. One of the participants discussed frustration about how a physician had alerted the child protective services immediately after she and her child left the hospital:

*I feel like if I was Norwegian it wouldn’t happen, but because we are different skin color from everybody, then people just think that immigrants come here without knowledge and they are not educated enough to understand things, and they beat their children and they do not have good homes. (P 19)*

She described how her child was intimidated by a blood test and kept crying, hiding himself under the physician’s table and saying that he did not want to go home. The participant believed that the physician had misinterpreted the child’s behavior; she explained that her child feared the needles badly since he had been through a lot of examinations. She was frustrated that the physician did not express her concerns or inform her about the decision to alert the child protective services:

*I feel like if I was Norwegian she would have spoken to me first, to say what is going on … because we came out, then I did not know what she is going to do. (P 19)*

This statement shows how the physician could have handled the situation differently by clarifying her concerns and making an informed decision.

## Discussion

This study reveals the experiences of immigrant parents of children with disabilities navigating the Norwegian healthcare system, particularly pediatric rehabilitation services. The aim of the study was to generate knowledge of how accessible and tailored the services were from the parents’ point of view. Although the findings of this study are derived from a Norwegian healthcare context, they may be relevant for understanding immigrants’ experiences elsewhere, because the Norwegian healthcare system is comparable to those in the Nordic and some other Western countries.

Previous studies of immigrant families have mostly explored their experiences of challenges while utilizing services. This study aimed to explore experiences of the services’ strengths as well as challenges. The immigrant parents were mainly satisfied with the follow-up services provided by the pediatric rehabilitation centers. Although participants’ satisfaction may reflect the quality of the health services provided to them [26], it is important to consider that these participants came from countries with very different services or even lacking a public welfare system. They had varied experiences of healthcare services in their home countries, which may have influenced their expectations, the way they experienced the Norwegian healthcare service, and their level of satisfaction [27]. Mangrio et al. [28] similarly showed how non-European parents felt gratitude when comparing the services received from child healthcare centers in Sweden with those in their countries of origin. They appreciated the way the service was organized and how well the children were cared for, the same way the parents in our study did. They expressed that the Swedish child healthcare system was good and that they could not find similar healthcare in their home countries. Czapka et al. [29] also noted how most of the Polish immigrants in their study compared the Polish and Norwegian healthcare systems and drew both positive and negative conclusions about the services provided to them in Norway.

Interestingly, a study of Turkish-speaking families of children with disabilities who were immigrants in the UK showed that parents appreciated the services they were given, even if they did not meet their expectations [30]. Sandhu et al. [30] interpreted these families’ appreciation as a reflection of their assumptions that health and social care support are privileges that can be withdrawn, rather than rights. This interpretation may also explain why some participants in our study were so grateful and hesitant to talk about the challenges they faced while navigating the services. Sandhu et al. [30] perceived that assumptions of the services as privileges, rather than rights, could also explain why immigrant families tended to respond to the challenges they faced with stoicism, which was less usual among non-immigrant families.

Despite their satisfaction with the follow-up services, participants in our study experienced several challenges while navigating the services. The challenges spontaneously shared were mostly about accessing help before their children were diagnosed. At that time, they felt that their concern had not been taken seriously by the healthcare providers and they did not receive the help they needed. A previous study conducted in the Netherlands also showed how mutual understanding and compliance is often worse in doctor–patient consultations with ethnic minority parents of pediatric patients than with their socially dominant counterparts [31]. That study suggested that the large differences in explanatory models of health and illness used by physicians and ethnic minority parents could be a reason for this poor mutual understanding. The same study found that consultations that ended without mutual agreement more often resulted in noncompliance with the prescribed therapy. As Van Wieringen et al. [31] stressed, healthcare providers’ communication skills when exploring the explanatory model with parents, and their open attitude to models other than a scientific medical paradigm, are important when interacting with immigrant families.

Eriksen et al. [32] also explained how some immigrants express their symptoms in a way that reflects their cultural background, which is minimally influenced by Norwegian medical thinking. These immigrants are unable to express themselves in a way that a Norwegian doctor is likely to take seriously. They struggle with accessing the treatment they need over a long period of time. Eriksen et al. [32] noted how Norwegian doctors and immigrant patients used the same word to describe two different conditions. According to them, in these cases, misunderstandings persisted partly because the doctors never tried to determine what the patient truly intended to say, and their conversation was not constructive.

The participants in our study also expressed unmet needs for information about their rights, the rules, and the services available to them and to which they were entitled. Sandhu et al. [30] found that although immigrant families of children with disabilities expressed gratitude for the services they were receiving in the UK, they felt that they were overlooked and not kept informed by service providers.

The experiences of needing information [33–36] and facing challenges due to a lack of access to timely help do not only affect immigrant families. Sloper et al. [37], in their study about the service needs of families of children with severe physical disabilities, showed how the difficulties in obtaining appropriate help caused these parents additional anxiety. Surprisingly, what Sloper et al. [37] noted in 1992 about the need for information, help in obtaining services, and a coordinated approach to providing services appropriate to all aspects of the family is still relevant and consistent with our findings.

Providing information about services and their availability is a crucial determinant for parents to take an active role in the care process, obtaining appropriate help and decision-making [37–39]. However, research has shown that healthcare providers are not always aware of the resources available to the families of children with disabilities [34, 40]. In addition, parents may find it difficult to define and express their information needs and may instead wait for healthcare providers to address a subject [41, 42]. These challenges explain how some participants in our study experienced accessing information by chance.

While providing information itself seems to be a challenge for service providers [43], immigrant parents in our study expressed the need for guidance and support in addition to information. Paperwork and the need for adequate writing skills, as well as knowing where to go or whom to contact, were challenges faced by several families in our study. Consistent with our findings, Fellin et al. [9] found that social workers acted as facilitators of services by assisting immigrant families in navigating the health and social systems. Interprofessional collaboration and connecting immigrant families with social workers is therefore important for their ability to manage navigating the health and social systems and connect with the appropriate resources [44].

Peer health navigator (PHN) interventions have also shown to be a promising approach to breaking down barriers to care for people from underserved populations, such as immigrants and ethnic minorities [45]. PHNs are individuals from the target population with shared lived experiences, and who have received specialized training to support and help others to navigate the complex and often fragmented healthcare delivery systems [45]. Providing PHN programs adapted to meet the needs of immigrant families of children with disabilities may therefore reduce barriers to their care, particularly for newly arrived families.

Navigating systems with multiple organizational and access issues has also been indicated as difficult for parents belonging to the majority of the population [33]. While these common challenges can complicate gaining access to and navigating the healthcare system for any family, certain challenges that are unique to immigrant families exacerbate the difficulties. Language barriers were noted by Lindsay et al. [40] as one of the main obstacles to accessing, receiving, and utilizing healthcare services for immigrant families of children with disabilities. Our findings show that language difficulties, issues with perceived interpreter quality, and issues of accuracy of translation are barriers to navigating the healthcare system [46, 47]. Therefore, while communicating with immigrant families in their nonprimary language or through an interpreter, healthcare providers should be aware of how this process may influence the quality of care and services provided to the families. It is the healthcare providers’ responsibility to ensure that their clients have understood the content of the conversation and to use a professional interpreter instead of relatives. Improvement of access to and use of professional interpreters is also important for both linguistic and cultural reasons [48].

The use of medical terminology [48, 49], lack of intercultural communication skills, and lack of training pertaining to working with families from diverse backgrounds were also communication challenges that immigrant families in our study faced. Healthcare providers may unintentionally devalue immigrant families’ perspectives and perceptions, assuming that the “Western way” is the “best and only way” [50]. According to Söderström [16], communication between healthcare workers and minority families occurs in the context of the healthcare providers’ perspectives and sense of reality. Healthcare providers need, therefore, to use culturally sensitive communication. This involves listening to and respecting the family, reflecting on their own knowledge and biases, and sharing their beliefs with the family [51]. Although ensuring equal access to public healthcare services for culturally diverse families of disabled children involves applying culturally sensitive communication [16], it is widely recognized that healthcare providers lack the skills to have culturally sensitive conversations with these families [52]. Therefore, enabling healthcare providers to use culturally sensitive communication by equipping them with the required knowledge and skills is important [51, 52].

Another important barrier to communication was the parents’ perceptions of the lack of adequate time to address their questions and concerns. Improving communication with immigrant families requires new and innovative solutions at the systemic level to provide enough time and/or make more efficient use of the time spent with these families, ensuring that their needs are met [40, 51]. A systematic review of 37 articles showed how the perceptions and practices of healthcare providers in providing services for immigrants were mainly influenced by cultural and language differences, as well as restricted institutional capacity in terms of time and/or resources [53]. King et al. [54] also noted how the context of the workplace can strongly affect the ability of therapists to deliver culturally sensitive care. They noted how the structured and time-limited therapeutic sessions and the organizational methods in the practice could restrict the time therapists had to get to know the family situation and build a collaborative relationship. However, it has been documented that time constraints also negatively affect ethnic majority patients’ experiences of communication in medical contexts and make them feel vulnerable. A systematic review of 57 qualitative studies targeting patients’ experiences in communicating with primary care physicians revealed negative experiences related to feeling vulnerable due to time constraints, regardless of patients’ ethnicity. Physicians were perceived to ask fewer questions or ask more closed questions, seemed disinterested, and tended to use jargon to rapidly explain the condition. Consequently, patients reported feeling dehumanized or “like a number”. Although experiences were similar among all patients, ethnic minority groups raised distinctive experiences relating to language barriers and differences in values and beliefs that further exerted a negative influence on their experience of communication [55].

Last, the findings of this study, in accordance with those of other research, show how perceived stereotypical attitudes towards immigrants can act as a barrier to these families navigating the healthcare system [29, 46]. Even the feeling of being treated differently than the majority may be experienced as offensive and can cause emotional distress among minority families. Such a sense of discrimination, in addition to other barriers related to the immigrant experience, can even cause immigrants to avoid using the healthcare system [29]. Thus, healthcare providers must be aware of their attitudes while interacting with people such as immigrants who have experienced many years of stigma and discrimination. This would be a step toward providing the services that are available and appropriate for the whole population, regardless of ethnicity, religion, function, or social status. A survey conducted at a Danish hospital showed that most healthcare providers considered immigrant patients to be a specific strain on the Danish healthcare system, which affected the attitudes towards immigrants of some providers. The same study showed that healthcare providers characterized some immigrant patients as being overly dramatic when ill [56].

As Penchansky et al. [57] noted, access to services is about the fit between the characteristics of providers and health services and the characteristics and expectations of users. Our findings show how the characteristics of healthcare providers and of immigrant families influence the families’ access to and utilization of the services. Therefore, practicing strategies that contribute to increasing the coherence between healthcare providers and immigrant families is important. Our findings also show how the discrepancy between the organization of services and the needs of immigrant families makes it challenging for families to use and navigate services. This underlines the importance of listening and responding to families’ perceptions of their own resources, challenges, and needs.

### Study strengths and limitations

While previous research conducted in Norway and internationally has mainly focused on particular minority populations, the participants in this study were immigrant parents with varied linguistic, cultural, and religious backgrounds. They came from different parts of Africa, Asia, and Eastern Europe, which together compose the largest immigrant group in Norway. There has also been little research exploring the experiences of immigrant families with pediatric rehabilitation services [9], as this research did.

It was not possible to parse out whether certain challenges were more likely to be reported by newer immigrant families, because this study did not include newly arrived families. The study included only immigrant families who had participated in an intensive pediatric rehabilitation program at a rehabilitation center. Applying for this program demands familiarity with the healthcare system and some language skills. Thus, the participants did not face the same challenges as newly arrived families might, and the results do not reflect the challenges and experiences of newly arrived families when seeking assistance from the healthcare system in Norway. However, the participants described their experiences of navigating the services during the very first years of their time in Norway, and those were included, analyzed, and reported in the results of this study.

Furthermore, member checking was not carried out. Norwegian is not the first language of the first author (interviewer) or the interviewees. This might have affected the quality of the produced data, despite the fact that the first author did her best to ensure a mutual understanding of the interview questions and the responses.

## Conclusion

This study was conducted to generate knowledge of how accessible and tailored the Norwegian healthcare system is from the perspective of immigrant parents of children with disabilities. The immigrant parents were mainly satisfied with the follow-up services provided by the pediatric rehabilitation centers, but they experienced several barriers while navigating the healthcare system. The barriers from the perspective of immigrant parents were associated with both the systemic and individual levels. At the individual level, the barriers were related to characteristics of both the families and the healthcare providers. Insufficient competence in the majority language, a lack of knowledge of the services, and a lack of understanding of how to navigate those services were some of the barriers related to family characteristics. Applying medical terms, a lack of experience and intercultural communication skills, and perceived attitudes were among the barriers related to the service providers’ characteristics. At the systemic level, the barriers were related to the use of interpreters who were perceived as being unprofessional, organizational routines, and insufficient time allotted for interactions with immigrant families. The lack of effective strategies to inform, empower, and enable immigrant families to manage navigating the complex and growing healthcare system was another barrier at the system level.

This study therefore highlights the importance of mobilization at both the individual and systemic levels. A need exists to reach out and educate immigrant groups to enable them to meet the complex demands of navigating the healthcare system in modern Norwegian society. Healthcare providers should particularly be aware of immigrants’ need to be informed and supported, even though they may not ask for it, so that they can make educated decisions while navigating the services. Healthcare providers should also be aware of the importance of effective communication and engagement and the impact of their interaction style on immigrant families of children with disabilities. Workplace teaching strategies and other training methods can be used to provide healthcare professionals with the training they need to develop the approaches needed to deliver care in a culturally sensitive manner.

This study adds to the current literature on the experience of accessing and utilizing healthcare services by showing how the immigrant experience affects the way the parents look at, experience, and appraise the services. The study reveals immigrant parents’ need for support in addition to information to manage navigating complex and changing services. The study also highlights the need for a coordinated approach to the assessment of need and the provision of services appropriate to all aspects of the family as a whole.

Our findings show that there is still a gap between the public ideal of equal healthcare services and the reality of the everyday lives of immigrant families of children with disabilities. The current gap challenges public policy on both the disability front and the immigrant front. By exploring the immigrant parents’ point of view, this study contributes to the opportunity to improve and adjust the services and the overall health and quality of life of these families and their children with disabilities.

Further research is needed to guide service providers and inform policymakers about the best ways of meeting the needs of immigrant families and their children with disabilities. Determining effective strategies for enhancing information transfer to immigrant families of children with disabilities is a relevant topic for future research. In addition, there is a need for research to develop PHN interventions adapted to meet the needs of immigrant families of children with disabilities, considering their success across diverse populations.

## Supplementary information

**Additional file 1.** Interview guide.

## Data Availability

The datasets generated and analyzed during the current study are not publicly available due to the need for participant anonymity but are available from the corresponding author on reasonable request.

## Abbreviations

CPOP: The national cerebral palsy surveillance program
GP: General practitioner
NSD: Norwegian Centre for Research Data
PHN: Peer health navigator
REK: Regional Committees for Medical and Health Research Ethics
UK: United Kingdom
US: United States
WHO: World Health Organization
