# Supplementary material for: Disabled and immigrant, a double minority challenge: a qualitative study about the experiences of immigrant parents of children with disabilities navigating health and rehabilitation services in Norway
Source: BMC Health Serv Res. 2020 Feb 22;20:134. doi: 10.1186/s12913-020-5004-2 (PMC7036199; doi:10.1186/s12913-020-5004-2)
Supplement: Supplementary file 1 — Additional file 1. Interview guide. [file 12913_2020_5004_MOESM1_ESM.docx]

**Interview guide**

1. Introduction

- Each interview begins with providing information about the interviewer, the project, interview aims, interview process and research ethics.

1. The experience of services in terms of both challenges and facilities

- How long have you been in touch with the healthcare system regarding the follow-up services of your child with disability within both the primary and specialist healthcare system?
- How available did you experience the services within both the primary and specialist healthcare system? Did you experience it as challenging to access the services that you needed? Or did you manage to access the services easily?
- What about the pediatric rehabilitation services? How available did you experience the pediatric rehabilitation services? How often did you receive the follow up services? Did you experience it as enough? If not, what did you do about that?
- How did you experience the pediatric rehabilitation services in terms of both challenges and facilities? What did you experience as the strengths of the services? What did you experience as limitations?
- Do you have any suggestion for improvement of the rehabilitation services in particular? What about the healthcare services in general, do you have any idea for improving the services?

1. The experience of interacting with the healthcare providers

- How did you experience your interaction in terms of relationship, cooperation and communication with the healthcare providers in general? Did you experience any challenges?
- How did you experience your interaction with healthcare providers within the pediatric rehabilitation services? How did you experience the relationship and cooperation with the healthcare providers?
- How did you experience the consultations with the pediatric rehabilitation team? Was it challenging to communicate with the healthcare providers? If yes, why did you experience it as challenging? And what did you do about that?
- Did you communicate with the healthcare provides through an interpreter? If yes, how did you experience communicating assisted by an interpreter?

1. The experience of receiving information

- Which kind of information did you usually receive from the healthcare providers? Did you experience it as useful?
- How did you experience receiving the information that you consider as important for your family or/and your child’s best?
- Do you experience that you receive the information that you need in order to navigate the services?

1. Reflective and ending questions

- Do you have any thoughts/reflections about why some of the healthcare providers experience it as challenging to reach out the immigrant families whom their children need services?
- Lastly, do you have any further comments that you want to add?
- May I (the interviewer) contact you again if I recognize that I have forgotten to ask you something, or something is unclear to me?
